# Activation of Nrf2-Regulated Glutathione Pathway Genes by Ischemic Preconditioning

**DOI:** 10.1155/2011/689524

**Published:** 2011-06-28

**Authors:** Karen F. S. Bell, Jill H. Fowler, Bashayer Al-Mubarak, Karen Horsburgh, Giles E. Hardingham

**Affiliations:** ^1^Centre for Integrative Physiology, University of Edinburgh, George Square, Edinburgh EH8 9XD, UK; ^2^Centre for Cognitive and Neural Systems, University of Edinburgh, Edinburgh EH8 9JZ, UK

## Abstract

Prophylactic pharmacological activation of astrocytic gene expression driven by the transcription factor Nrf2 boosts antioxidant defences and protects against neuronal loss in ischemia and other disease models. However, the role of Nrf2 in mediating endogenous neuroprotective responses is less clear. We recently showed that Nrf2 is activated by mild oxidative stress in both rodent and human astrocytes. Moreover, brief exposure to ischemic conditions was found to activate Nrf2 both *in vivo* and *in vitro*, and this was found to contribute to neuroprotective ischemic preconditioning. Here we show that transient ischemic conditions *in vitro* and *in vivo* cause an increase in the expression of Nrf2 target genes associated with the glutathione pathway, including those involved in glutathione biosynthesis and cystine uptake. Taken together, these studies indicate that astrocytic Nrf2 may represent an important mediator of endogenous neuroprotective preconditioning pathways.

## 1. Introduction

### 1.1. Nrf2 Is a Master Regulator of Antioxidant Gene Expression

 Many acute and chronic neurological disorders are associated with oxidative stress, caused by an imbalance in the production and detoxification of reactive oxygen species (ROS). Nuclear factor-erythroid 2-related factor 2 (Nrf2), a member of the cap“n”collar transcription factor family, is a master regulator of antioxidant defense genes and drug-metabolizing enzymes [1, 2]. The binding of Nrf2 to a *cis*acting DNA promoter sequence, called the antioxidant response element (ARE), allows transactivation of a group of cytoprotective genes [1, 2]. Under normal conditions Nrf2 is bound to Kelch-like ECH-associated protein 1 (Keap1), and through a two-site interaction, the transcription factor is ubiquitinated by Cul3/Rbx1 and targeted for degradation [3]. However, under oxidative stress conditions the two-site interaction between Nrf2 and Keap1 is disrupted, allowing Nrf2 to evade Keap1-mediated ubiquitination and accumulate in the nucleus where it activates genes with an ARE sequence within their promoters, leading to an induction of antioxidant machinery [2]. Upregulation of the ARE-gene battery has a significant impact on the ability of the cell to withstand and survive sustained oxidative insults. Prophylactic Nrf2 activation by small molecules is protective against a host of oxidative insults *in vitro*, including free radical donors and oxygen glucose deprivation (OGD), as well as toxic levels of glutamate or N-methyl-D-aspartate (NMDA, [4–6]). Nrf2 activation *in vivo* is similarly protective, reducing neurotoxin or stroke-induced injury [5, 7, 8].

### 1.2. Nrf2-Mediated Neuroprotection in the CNS

Nrf2 has an integral role in mediating antioxidant responses within the CNS. Nrf2-dependent gene expression in astrocytes can protect neurons against a variety of trauma and disease-causing agents [9–14]. In these studies, however, Nrf2-dependent gene expression was achieved artificially, either via astrocyte-specific overexpression of Nrf2, or through treatment with well-characterized small molecule activators of the pathway. The latter is in essence a form of chemoprevention, whereby prophylactic administration of small molecule Nrf2 activators confers significant neuroprotection [4, 5, 7, 15]. Indeed, fibroblasts are also rendered resistant to many electrophiles, peroxides, and redox-cycling agents, in an Nrf2-dependent fashion, by pretreatment or “priming,” with nontoxic doses of chemopreventive agents such as sulforaphane [16]. Prophylactic administration of Nrf2 activators can be considered an artificial pharmacological form of preconditioning, whereby exogenous activation of the antioxidant response renders the cell better able to defend itself from a subsequent insult. However, little was known as to what *endogenous* signals might trigger an Nrf2-dependent, physiologically relevant, endogenous, neuroprotective response.

## 2. Results and Discussion

### 2.1. Ischemia and Oxidative Stress Activate Nrf2 in Astrocytes

In nonneural cells, oxidative stress is known to activate Nrf2 through inhibition of Keap1-dependent Nrf2 degradation. This is effected at least in part via the modification of key cysteine residues in Keap1 and may result in the formation of disulfide bonds [17] and possibly conformational changes in the adaptor protein [2]. To investigate whether oxidative stress activates Nrf2 in neural cells, we chose two experimental models which recreate this *in vitro*, OGD and H_2_O_2_ application [18]. Exposure times or concentrations were selected, which bridged both lethal and sublethal doses in order to confirm oxidative-stress-dependent Nrf2 activation in physiologically relevant, viable conditions. Peroxide is a ROS with clearly identified roles in contributing to oxidative stress, while OGD and subsequent reoxygenation trigger a complex cascade of detrimental events including excitoxicity, cytosolic and mitochondrial ROS generation, and inflammatory responses (for review see [19]). Anoxia causes an accumulation of reducing equivalents within the electron transport chain, which upon reperfusion triggers a burst of ROS production [20]. Oxidative stress and ROS production also arise from impaired glutamate signaling due to excessive transmitter release and a reversal of glutamate uptake transporters. As a result excessive NMDAR activity triggers superoxide production via NADPH oxidase activation and xanthine oxidase [21]. 

We found that both OGD and mild oxidative stress (subtoxic H_2_O_2_) applied to mixed neuronal/astrocytic cultures [18] induced expression of Nrf2 target genes [18], including the classical target gene, *hemeoxygenase *(*Hmox1*) and the more recently identified target gene, *sulfiredoxin *(*Srxn1*). Given that the *Srxn1* and *Hmox1* genes can respond to factors other than Nrf2 [22], whether Nrf2 was the specific transcription factor mediating the gene induction following ischemia or H_2_O_2_ application remained unclear. To determine this, the same protocols were employed on Nrf2 −/− cultures revealing no significant gene induction after OGD or after H_2_O_2_ application in the absence of Nrf2, highlighting the central role of endogenous Nrf2 in mediating this gene induction [18]. Of note, we found that the locus of Nrf2 activation within the mixed cultures is centred on the astrocytes. Induction of Nrf2 target genes by OGD or oxidative stress was not observed in pure neuronal cultures devoid of astrocytes, while activation in pure astrocytic cultures was found to be highly robust [18]. Immunofluorescent analysis in mixed cultures of *Hmox1* induction by oxidative stress also revealed induction that was specific to GFAP-positive astrocytes [18].

### 2.2. Astrocytic Nrf2 Contributes to Neuroprotective Ischemic Preconditioning

 We next assessed whether the increase in Nrf2-regulated gene expression translated into enhanced protection, by quantifying neuronal viability following OGD in Nrf2 −/− and wild-type mixed cultures. Following OGD no difference in survival was observed between the Nrf2 wild-type and −/− neurons [18], which was perhaps surprising given the known protective capacity of Nrf2, although easily explained by the fact that Nrf2 target gene induction by this toxic insult was arising too late to confer protection. We next hypothesized that a sublethal insult might be sufficient to activate Nrf2, potentially contributing to neuroprotective ischemic preconditioning [23]. We established a preconditioning protocol, where a nontoxic 1.5 h exposure to OGD conferred significant neuroprotection against a toxic 3 h OGD insult applied 24 h later [18]. To determine whether the initial 1.5 h OGD insult led to recruitment of the Nrf2 pathway, Nrf2-dependent gene expression was assessed following the preconditioning stimulus. The 1.5 h OGD preconditioning stimulus led to a significant upregulation in both *Srxn1* and *Hmox1* gene expression, demonstrating activation of the Nrf2 pathway [18]. This suggested a possible contribution of astrocytic Nrf2 activation in the neuroprotection incurred by our preconditioning protocol.

To determine the role of Nrf2 in the protective effect of ischemic preconditioning, Nrf2 wild-type and −/− mixed cultures were exposed to the preconditioning protocol. While a preconditioning-induced increase in viability was apparent in both Nrf2 wild-type and −/− neurons, the magnitude of the protective effect of preconditioning was significantly diminished in the absence of Nrf2 [18]. Preconditioning reduced the total amount of OGD-induced death by around 60% in wild-type neurons, but by under 30% in Nrf2-deficient neurons [18]. Thus, Nrf2 activation is responsible for a significant portion of the protective effect of ischemic preconditioning *in vitro*. We next determined whether the Nrf2 pathway might also be implicated in *in vivo* preconditioning. To achieve this, Nrf2 target gene expression was assessed in mice subjected to a 15-minute occlusion of the middle cerebral artery (MCA), a stimulus known to trigger preconditioning and confer significant protection from subsequent ischemic episodes *in vivo* [24]. Cortical extracts from the ipsilateral hemisphere revealed a significant upregulation of both *Srxn1* and *Hmox1 *following transient occlusion [18], demonstrating that an ischemic preconditioning episode *in vivo* activates the Nrf2 pathway.

### 2.3. Activation of Nrf2-Regulated Glutathione Pathway Genes by Preconditioning Stimuli

 In addition to *Srxn1* and *Hmox1*, Nrf2 also regulates the expression of enzymes involved in glutathione synthesis and utilisation, a system which contributes substantially to cells' antioxidant capacity. Indeed, activation of Nrf2 in astrocytes by overexpression of small molecules is thought to promote neuroprotection due to the coordinated upregulation of glutathione synthesis and release, leading to increased availability of glutathione precursors for nearby neurons to use to enhance their own glutathione pool [25].

**Figure 1:**
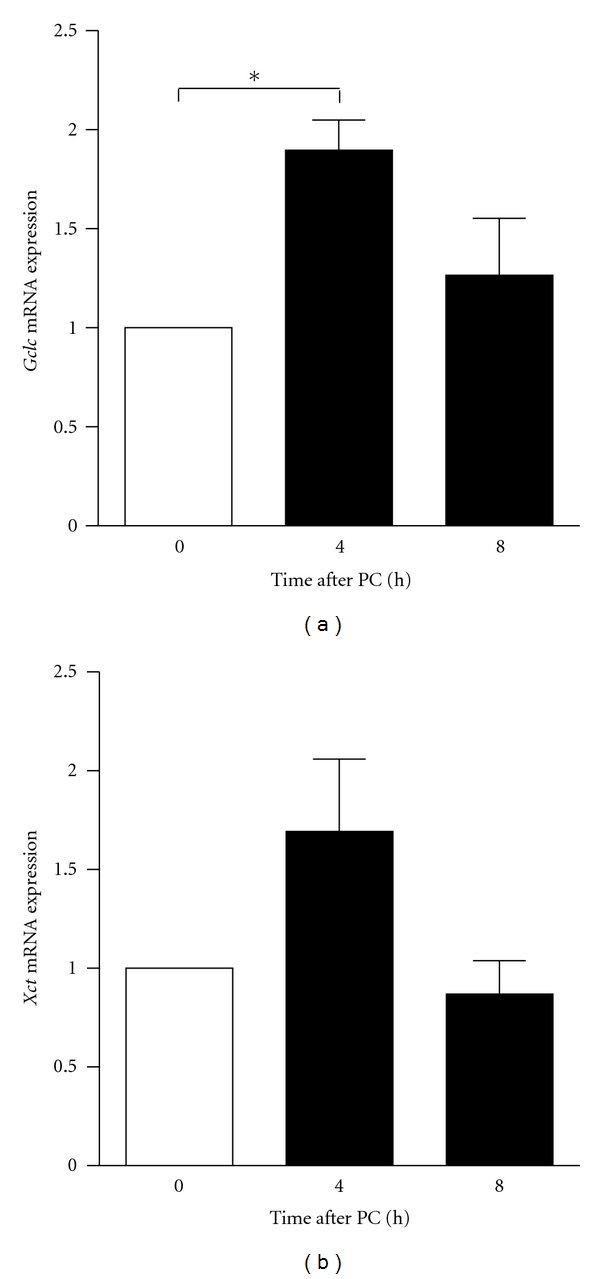
*In vitro* preconditioning activates *Gclc* expression. neurons were exposed to the 1.5 h OGD preconditioning stimulus and the expression of Nrf2-target genes. *Gclc* and *xCT *were determined by qPCR. Expression of both *xCT *and* Gclc *was significantly upregulated by the preconditioning stimulus, highlighting a potential contribution of their gene products in the neuroprotection acquired by preconditioning (bars represent mean ± SEM, *P < .05, unpaired *t*-test, *n* = 3 for *Gclc*, *n* = 5 for *xCT*).

**Figure 2:**
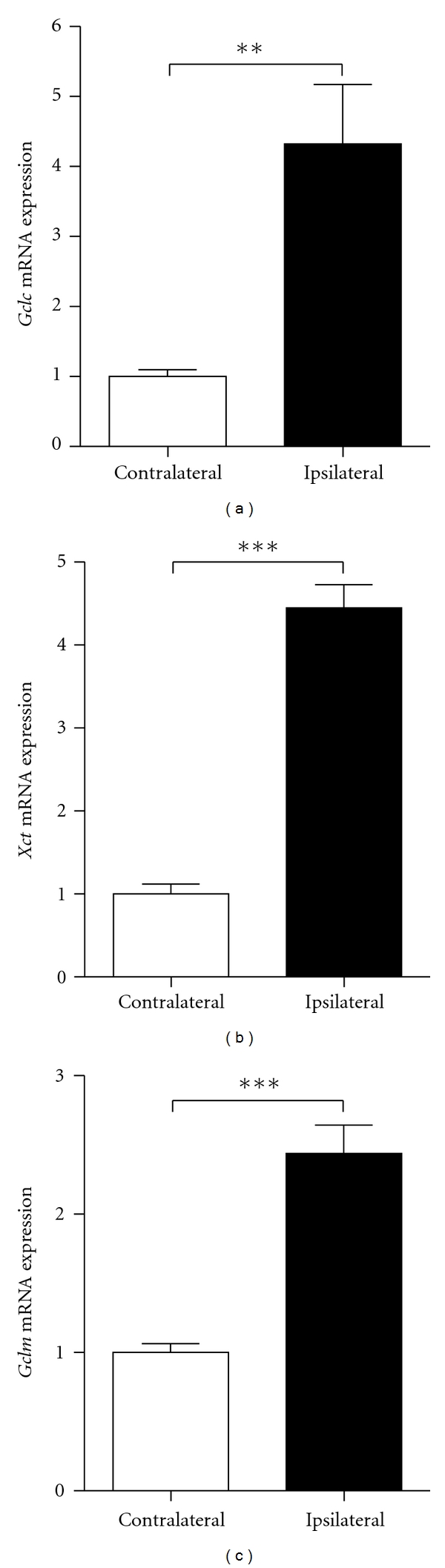
Upregulation of glutathione pathway components following *in vivo* ischemic preconditioning. Gene expression of glutathione pathway components,* Gclc *(a),* xCT *(b), and *Gclm* (c), was quantified by qPCR in mice subjected to a transient 15-minute occlusion of the middle cerebral artery, a stimulus known to trigger neuroprotective preconditioning *in vivo*. Transient ischemia significantly increased the cortical expression of *Gclc*, *xCT*, and *Gclm* in the ipsilateral hemisphere, as compared to the contralateral hemisphere, suggesting a specific recruitment of the ARE/Nrf2/glutathione pathway in the protective effects of preconditioning *in vivo* (bars represent mean ± SEM,**P < .01, ***P < .001, Student's *t*-test, *n* = 6).

To determine whether glutathione pathway genes are induced following preconditioning, gene expression of representative Nrf2-controlled glutathione pathway members were quantified by quantitative real-time PCR (qPCR) 4 or 8 h following exposure to the 1.5-hour OGD preconditioning stimulus. Expression of *glutamate-cysteine ligase* (*Gclc*) and *glutamate/cystine antiporter* (*xCT*) was assessed; *Gclc* encodes the catalytic subunit of the rate-limiting enzyme of glutathione synthesis, and xCT encodes a cystine/glutamate transporter and represents the major mechanism for obtaining cysteine for glutathione synthesis. Exposure to the ischemic preconditioning stimuli conferred a significant increase in *Gclc* gene expression (Figure 1(a), *P < .05, unpaired *t*-test, *n* = 3) and a near significant increase in *xCT* expression 4 h following OGD (Figure 1(b), *P* = .06, unpaired *t*-test, *n* = 5). To assess whether recruitment of the glutathione pathway might also occur *in vivo*, adult mice were subjected to a 15-minute occlusion of the MCA, which as mentioned above is a stimulus known to trigger *in vivo* preconditioning. Four hours later, mice were sacrificed and cortical tissue was harvested from the ipsilateral and contralateral hemispheres for RNA isolation and qPCR. Transient occlusion of the MCA triggered a significant upregulation in the mRNA expression of both *Gclc *and *xCT* in the ipsilateral cortex (see Figures 2(a) and 2(b), **P < .01, ***P < .001, Student's *t*-test, *n* = 6), as well as in the expression of another Nrf2 target gene, *Glutamate-cysteine ligase regulatory subunit* (*Gclm*) (Figure 2(c), ***P < .001, Student's *t*-test, *n* = 6), suggesting a potential contribution of these gene products to the resultant neuroprotection acquired by ischemic preconditioning* in vivo*. Thus, a variety of Nrf2 target genes are induced by transient ischemia *in vivo*. 

### 2.4. Nrf2-Dependent and Nrf2-Independent Mechanisms of Astrocyte-Mediated Neuroprotection

 Our finding that astrocytic Nrf2 plays a role in preconditioning is in line with studies identifying ROS production as a necessary event in the establishment of preconditioning [26] and the finding that superoxide radicals or H_2_O_2_ is capable of activating the preconditioning response [27]. Moreover, our finding that the gene expression of key markers of the glutathione pathway is increased following *in vitro* and *in vivo* preconditioning supports a neuroprotective role for the glutathione pathway in the Nrf2-dependent component of ischemic preconditioning. Indeed, numerous studies have identified glutathione as being involved in preconditioning [28, 29], and chemopreventive protection is associated with a marked upregulation of glutathione biosynthesis [16], as is Nrf2 activation in the brain [7, 15]. 

In contrast to our own findings, a recent study identified an Nrf2-independent neuroprotective effect following subtoxic H_2_O_2_ generation in astrocytes [30]. The authors utilized an elegant system whereby the expression of a H_2_O_2_-producing enzyme in astrocytes led to a specific and quantifiable level of H_2_O_2_ production following application of D-alanine. This in turn led to a protective response in astrocytes that rendered neurons resistant to an oxidative insult. However, this response was not dependent on astrocytic Nrf2 activation and potentially involved tyrosine phosphatase inhibition [30]. While the potential reasons behind the disparities between the two studies are discussed elsewhere [18], it is feasible that both Nrf2-dependent and Nrf2-independent mechanisms can contribute to adaptive neuroprotective responses by astrocytes to mild oxidative insults. Moreover, the importance of individual pathways may depend on developmental stage or the severity or nature of the oxidative insult. Notwithstanding these issues, it is clear that astrocytes are important mediators of adaptive neuroprotective responses to subtoxic insults.

### 2.5. Concluding Remarks

Our identification of astrocytic Nrf2, a mediator of endogenous ischemic preconditioning, underlines the importance of astrocytes in shaping neuronal vulnerability to insults. Moreover it emphasizes the potential value of astrocytic Nrf2 as a therapeutic target in a variety of disorders associated with oxidative stress [31]. Since Nrf2 controls multiple components of both the glutathione system and the thioredoxin-peroxiredoxin system [2, 31, 32], it has the capacity to mount a coordinated antioxidant response to oxidative insults in the brain. While studies have been focussed on rodent systems, it will be important to determine whether human astrocytes are capable of mediating a neuroprotective response to Nrf2-activating stimuli, something that human stem-cell-based approaches are now capable of answering.

## 3. Materials and Methods

### 3.1. Neuronal Cultures

Cortical mouse mixed cultures of neurons and astrocytes were prepared as described [33] from E17.5 CD1 mice with neurobasal growth medium supplemented with B27 (Invitrogen, Carlsbad, Calif, USA). These cultures involve approximately 90% NeuN-positive neurons and 10% GFAP-positive astrocytes [34]. Experiments were carried out on cultured neurons following a period of 8–10 days during which cortical neurons develop a network of processes, express functional NMDA-type and AMPA/kainate-type glutamate receptors, and form synaptic contacts. Prior to the start of experiments, neurons were subjected to trophic deprivation by transferring them from growth medium to TMo for two hours, a medium containing 10% MEM (Invitrogen) and 90% salt-glucose-glycine (SGG) medium (SGG: 114 mM NaCl, 0.219% NaHCO_3_, 5.292 mM KCl, 1 mM MgCl_2_, 2 mM CaCl_2_, 10 mM HEPES, 1 mM glycine, 30 mM glucose, 0.5 mM sodium pyruvate, 0.1% phenol red; osmolarity 325 mosm/l, [35]).

### 3.2. Oxygen Glucose Deprivation

OGD was performed on DIV = 9 cultured mouse neurons. Cells were transferred from TMo, washed once in a glucose-free, balanced salt solution (SGG with mannitol substituted for glucose, SGG-Mann): 114 mM NaCl, 0.219% NaHCO_3_, 5.292 mM KCl, 1 mM MgCl_2_, 2 mM CaCl_2_, 10 mM HEPES, 1 mM glycine, 30 mM mannitol, 0.5 mM sodium pyruvate, 0.1% phenol red; osmolarity 325 mosm/l solution, which had previously been degassed by flushing the solution with 95% N_2_-5% Co_2_ for 30 min Cells were placed in degassed glucose-free SGG-Mann and put in a modular incubator chamber, which was flushed with 95% N_2_-5% Co_2_ for 4 min at a flow rate of 20 L/min, according to manufacturer's instructions (Billups-Rothenburgh, Del Mar, Calif, USA), in order to fully expel any remaining oxygen within the chamber. The chambered cells were then left in OGD at 37°C for 3 h, before being returned to normoxic conditions and glucose-containing media (TMo). No OGD control cells were placed in SGG and maintained in normoxic conditions for 3 h before also being returned to TMo. All cells were left in TMo until RNA isolation for the time point indicated. Anaerobic conditions within the modular incubator chamber were confirmed with Dry Anaerobic Indicator Strips (Fisher Scientific, Loughborough, UK).

### 3.3. RNA Isolation, RT-PCR, and qPCR

RNA was isolated using the Stratagene Absolutely RNA Miniprep kit as directed by the manufacturer, including the optional DNAse treatment (Stratagene, Amsterdam, Netherlands). For qPCR, cDNA was synthesized from 1–3 *μ*g RNA using the Stratascript QPCR cDNA Synthesis kit (Stratagene) according to the manufacturer's instructions and as described previously [6, 34]. Briefly, the required amount of RNA (up to 3 *μ*g) was diluted in RNase-free water (up to 7 *μ*L final volume) and mixed on ice with 2x cDNA Synthesis master mix (10 *μ*L), random primers: oligo-dT primers 3:1 (total 2 *μ*L–200 ng), and either 1 *μ*L RT/RNase block enzyme mixture (for RT reactions) or 1 *μ*L water (for no-RT control reactions). Reaction mixtures were mixed, spun down, and incubated for 2 min at 25°C, 40 min at 42°C, and 5 min at 95°C. cDNA was stored at −20°C. Dilutions of this cDNA were subsequently used for real-time PCR (cDNA equivalent to 6 ng of initial RNA per 15 *μ*L qPCR reaction for all genes). qPCR was performed in an Mx3000P qPCR System (Stratagene) using Brilliant SYBR Green qPCR Master Mix (Stratagene) according to the manufacturer's instructions. Briefly, the required amount of template was mixed on ice with 2x Brilliant SYBR Green Master Mix, forward and reverse primers at 200 nM each final concentration, 30 nM final concentration ROX passive reference dye, and water to the required reaction volume. Technical replicates as well as no-template and no-RT negative controls were included, and at least 3 biological replicates were studied in each case. The sequence of the utilized primers is as follows (all at 200 nM final): *xCT-F*: 5′-ATACTCCAGAACACGGGCAG-3′, *xCT-R*: 5′-AGTTCCACCCAGACTCGAAC-3′, *Gclc-F*: 5′-CCAACCATCCGACCCTCTG-3′, *Gclc*-*R*: 5′-TGTTCTGGCAGTGTGAATCC-3′, *Gclm-F*: 5′-GCACAGCGAGGAGCTTC-3′, *Gclm-R*: 5′-GAGCATGCCATGTCAACTG-3′, *GAPDH-F*: 5′-GGGTGTGAACCACGAGAAAT-3′, *GAPDH-R*: 5′-CCTTCCACAATGCCAAAGTT-3′. The qPCR cycling programme was 10 min at 95°C; 40 cycles of 30 sec at 95°C, 40 sec at 60°C with detection of fluorescence, and 30 sec at 72°C; 1 cycle (for dissociation curve) of 1 min at 95°C and 30 sec at 55°C with a ramp up to 30 sec at 95°C (ramp rate: 0.2°C/sec) with continuous detection of fluorescence on the 55–95°C ramp. Data were analysed using the MxPro qPCR analysis software (Stratagene), and the expression of the gene of interest was normalized to GAPDH, a commonly used control.

### 3.4. *In Vivo* Focal Cerebral Ischemia

All experiments were carried out in adult male C57Bl/6J mice (Charles River, UK) under an appropriate Home Office Licence and adhered to regulations as specified in the Animals (Scientific Procedures) Act (1986). Transient focal ischemia (15 min) was induced by intraluminal filament occlusion of the right middle cerebral artery (MCA). Animals were anaesthetized and maintained with isoflurane (2%) in a mixture of 30% O_2_ and 70% N_2_O by face mask. Focal cerebral ischemia was induced by the occlusion of the right MCA with an 8–0 nylon monofilament (Ethicon, Kirkton, Scotland) coated with a mixture of silicone resin (Xantoprene, Bayer Dental, Osaka, Japan) and hardener (Elastomer Activator, Bayer Dental). Briefly, the right common carotid (CCA), external carotid (ECA), and internal carotid (ICA) arteries and their branches were exposed through a midline cervical incision. A 6–0 silk suture was tied around the CCA proximal to the bifurcation of the ECA and ICA and then a second suture tied around the ECA distal to the superior thyroid artery (STA). The STA and occipital artery (OA) were closed by electrocoagulation. The silicone-coated monofilament (diameter 220 *μ*m) was introduced into the CCA via a small incision and advanced 10 mm distal to the carotid bifurcation so as to occlude the MCA. Following the occlusion, mice were then recovered from anaesthesia briefly and placed in an incubator (30°C) before being reanesthetized in order to remove the monofilament to allow reperfusion. Wounds were sutured closed and anaesthesia discontinued. After reperfusion of 3.75 h (*n* = 6), mice were reanesthetized briefly with 5% isoflurane and decapitated. The brains were rapidly removed, and the regions supplied by the MCA (striatum and cortex) were dissected within the ipsilateral and contralateral MCAO territory, frozen in liquid nitrogen, and kept at −80°C for subsequent RNA analysis. Frozen tissue samples (ipsi- and contralateral) were weighed and homogenised in a 1 ml glass Dounce homogeniser, and RNA was isolated as described above using the Stratagene Absolutely RNA mini prep kit (Stratagene). Sham-operated animals were treated as above except that no occlusion of the MCA was performed.
